# Influence of heme and pH on the oligomeric states of *Brucella melitensis* bacterioferritin

**DOI:** 10.1107/S2059798326006625

**Published:** 2026-08-10

**Authors:** Tanguy Scaillet, Clément Champagne, Johan Wouters

**Affiliations:** a Department of Chemistry, Laboratoire de Chimie Biologique Structurale (CBS) University of Namur (UNamur), Namur Research Institute for Life Sciences (NARILIS), Namur Institute of Structural Matter (NISM) 61 Rue de Bruxelles 5000Namur Belgium; University of Queensland, Australia

**Keywords:** bacterioferritin, *Brucella melitensis*, oligomerization, interfaces, crystallization, thermal stability

## Abstract

Bacterioferritin from *Brucella melitensis* (*Bm*Bfr) is a protein that plays essential roles in oxidative stress management and metal homeostasis in the bacterium. It presents itself as a 24-mer forming a spherical nanocage, allowing further applications such as drug delivery and nanoparticle synthesis. Studying the oligomerization process is thus important for a deeper understanding of the protein. In this work, two forms of the protein were studied: with the heme cofactor (holo *Bm*Bfr) and without the cofactor (apo *Bm*Bfr). Analyses were performed using size-exclusion chromatography, thermal shift analysis and structure determination of apo *Bm*Bfr by X-ray crystallography. This revealed that oligomerization is not possible with the apo form, since the heme is essential for dimer formation, which is the first oligomerization step. Further oligomerization can occur at a pH above 5.0, where interface interactions are favorable, to form a highly stable 24-mer state. Those findings allowed us to suggest an oligomerization mechanism for *Bm*Bfr.

## Abstract 2

The oligomerization of *B. melitensis* bacterioferritin (*Bm*Bfr) was studied using X-ray crystallography, size-exclusion chromatography and thermal shift analysis. These techniques allowed the characterization of both apo and holo *Bm*Bfr, highlighting the impact of heme and pH on the oligomerization mechanism.

## 1. Introduction

*Brucella* species are facultative intracellular pathogens responsible for brucellosis, a zoonosis targeting mammals, and are thus of major public health and economic importance in many developing regions (Laine *et al.*, 2023 ▸; Lokamar *et al.*, 2020 ▸; Kiiza *et al.*, 2023 ▸). *Brucella* are α-proteobacteria which infect diverse cell types, including phagocytes, where they replicate while avoiding host immune defenses (Ackermann *et al.*, 1988 ▸; Corbel *et al.*, 2006 ▸; Moreno, 2014 ▸). To achieve this, they have evolved mechanisms that disrupt dendritic cell functions, thereby avoiding detection by the host immune system (Lacerda *et al.*, 2013 ▸; Salcedo *et al.*, 2008 ▸). The different species of *Brucella* that exist differ in their membrane structure and secretion systems, which may contribute to their distinct host preferences (Eschenbrenner *et al.*, 2006 ▸; Velasco *et al.*, 2000 ▸).

Survival within macrophages requires *Brucella* to withstand profound iron limitation and high levels of oxidative stress. Under these intracellular conditions, efficient management of iron becomes essential to maintain metabolic activity while preventing the iron-driven formation of reactive oxygen species (Baldwin & Roop, 2002 ▸).

**Figure 1:**
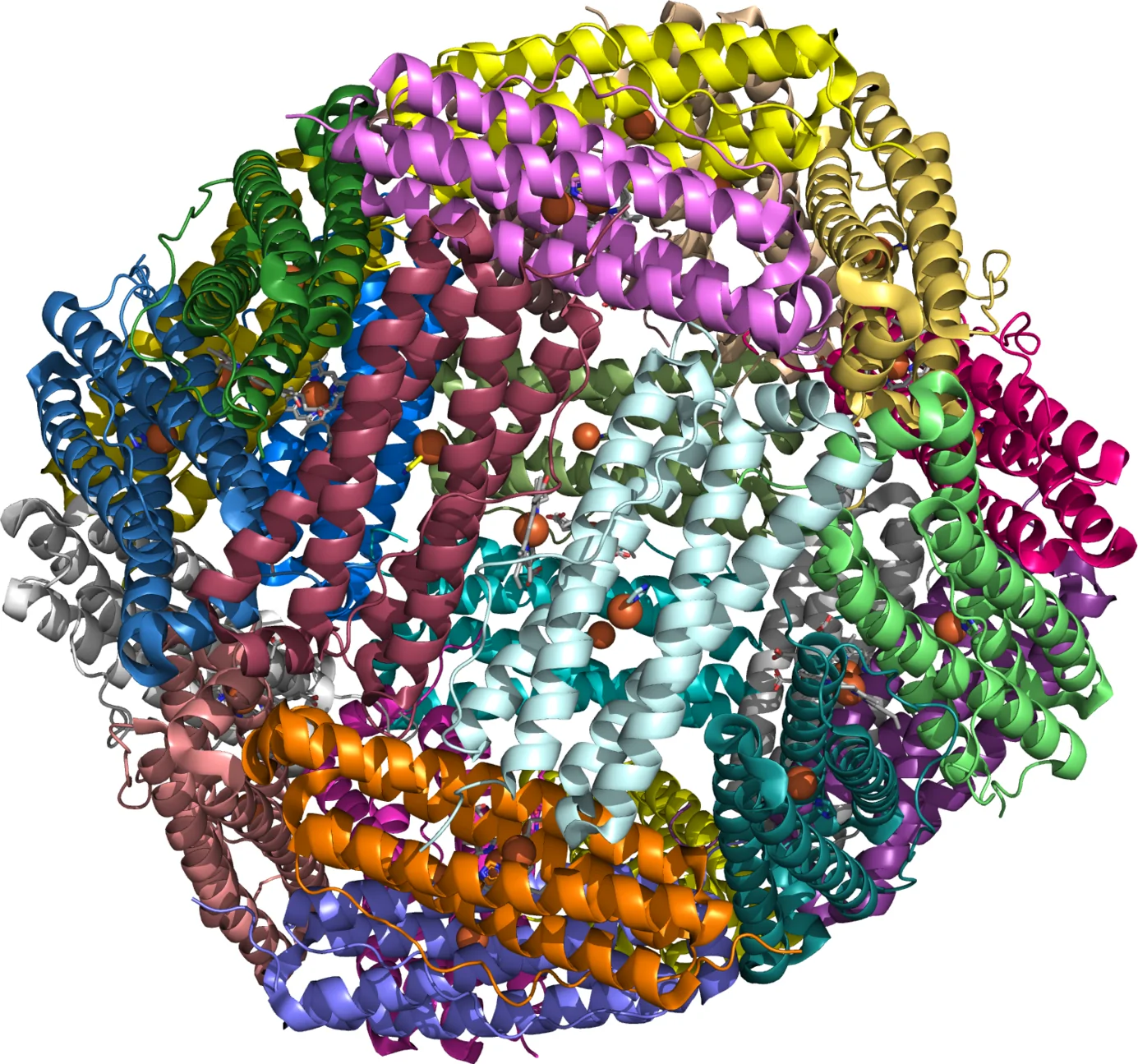
Structure of *B. melitensis* bacterioferritin (*Bm*Bfr, PDB entry 3fvb).

Ferritin-like proteins such as bacterioferritin (Bfr; Fig. 1 ▸) are therefore central to *Brucella* pathophysiology, playing essential roles in oxidative stress management and metal homeostasis in bacteria. Bacterioferritins (Bfrs) belong to the ferritin superfamily and serve as key iron-storage and detoxification systems in many bacteria (Almirón & Ugalde, 2010 ▸; Arosio *et al.*, 2009 ▸; Crow *et al.*, 2009 ▸). They assemble into a highly conserved homo-24-mer protein nanocage with a large internal cavity that mineralizes Fe^3+^, thereby preventing the accumulation of redox-active Fe^2+^. Each subunit adopts a characteristic four-helix-bundle fold containing a di-iron ferroxidase center. Mechanistic studies in *Escherichia coli* have defined the canonical ferroxidation pathway: two Fe^2+^ ions bind the catalytic site, react with O_2_ or H_2_O_2_ to generate a di-Fe^3+^ intermediate and undergo turnover supported by a tyrosine-based electron-transfer network (Bradley *et al.*, 2014 ▸, 2015 ▸, 2024 ▸). Other mechanistic studies in *Pseudomonas aeruginosa* showed that a second protein, the bacterioferritin-associated ferredoxin (Bfd), is involved in the transport of iron ions to the ferroxidase center, forming a Bfr–Bfd complex structure (Weeratunga *et al.*, 2010 ▸; Yao *et al.*, 2012 ▸). This model can be extrapolated to *B. melitensis* bacterioferritin (*Bm*Bfr), which possesses a highly conserved structure (Liu *et al.*, 2026 ▸).

Despite only 48.4% sequence identity between *Bm*Bfr and *E. coli* Bfr, and 21.1% identity between *Bm*Bfr and human ferritin, ferritin-like proteins consistently retain the 24-mer assembly and overall structural design (Andrews *et al.*, 1989 ▸; Costanzo *et al.*, 1984 ▸; Denoel *et al.*, 1995 ▸; van Eerde *et al.*, 2006 ▸; Wang *et al.*, 2006 ▸). Understanding the oligomeric state of bacterioferritins is thus crucial for interpreting how they control iron flux. Previous studies in other organisms suggest that the ferritin-assembly process occurs spontaneously, with the dimer as the first stable intermediate (Ebrahimi *et al.*, 2015 ▸). It subsequently oligomerizes into a final 24-meric cage. Bacterio­ferritin differs from other ferritins in that it contains up to 12 heme groups per 24-mer (Andrews *et al.*, 1995 ▸; Crow *et al.*, 2009 ▸). These heme groups are bound at the dimeric interface, deep below the protein surface, and coordinate to a methionine residue in each monomer (Mohanty *et al.*, 2021 ▸; Rivera, 2017 ▸; Wang *et al.*, 2015 ▸; Wong *et al.*, 2009 ▸). Studies on *E. coli*, *Mycobacterium tuberculosis* and *P. aeruginosa* indicate that the heme group is mainly involved in structural stability and facilitates electron transfer and iron release from the bacterioferritin core (Le Vay *et al.*, 2020 ▸; Mohanty *et al.*, 2021 ▸; Pullin *et al.*, 2021 ▸; Wang *et al.*, 2015 ▸) but is not essential for the oligomerization process (Andrews *et al.*, 1995 ▸; Mohanty *et al.*, 2021 ▸; Parida *et al.*, 2025 ▸).

Oligomerization is often modulated by physicochemical conditions, including pH, ionic strength, temperature and ligand binding (Ali & Imperiali, 2005 ▸; Gaber & Pavšič, 2021 ▸; Liu & Eisenberg, 2002 ▸). These transitions can directly affect ferritin-pore dynamics, ferroxidase cooperativity and metal-trafficking pathways.

The capacity of ferritin nanocages to assemble, disassemble and be engineered has inspired applications ranging from targeted drug delivery and imaging to confined nanoparticle synthesis and protein encapsulation (Johnson-Arbor & Dubey, 2023 ▸; Mohanty *et al.*, 2022 ▸; Saldan *et al.*, 2015 ▸; Song *et al.*, 2021 ▸; van der Ven *et al.*, 2023 ▸; Wang *et al.*, 2011 ▸; Zheng *et al.*, 2009 ▸; Zhen *et al.*, 2013 ▸).

Here, we report structural characterization of *Bm*Bfr combining size-exclusion chromatography (SEC) and X-ray crystallography (XRD). SEC is used to define its oligomeric state and solution behavior at different pH values, while XRD was performed on apo *Bm*Bfr to provide a high-resolution description of its quaternary assembly and ferroxidase center and compare it with the holo *Bm*Bfr structure (PDB entry 3fvb; Seattle Structural Genomics Center for Infectious Disease. unpublished work). Particular attention is given to the effect of the heme cofactor in *Bm*Bfr assembly. While heme is not considered to be essential for oligomerization in other bacterioferritins, our observations suggest that its contribution in *B. melitensis* may differ. We therefore investigate the structural and oligomeric consequences of heme binding by comparing apo and holo *Bm*Bfr.

## 2. Materials and methods

### 2.1. Enzyme overexpression and purification

*B. melitensis* bacterioferitin was overexpressed after induction with isopropyl β-d-1-thiogalactopyranoside (IPTG) in *E. coli* cells (BL21pLysS strain) containing a plasmid coding for the His-tagged protein (BrabA.00028.a) provided by the Seattle Structural Genomics Center for Infectious Disease (https://www.ssgcid.org), which is supported by Federal Contract No. 75N93022C00036 from the National Institute of Allergy and Infectious Diseases, National Institutes of Health, Department of Health and Human Services. Bacteria were grown in liquid lysogeny broth medium (LB) at 37°C until an optical density of 0.6 at 600 nm was reached, after which induction was performed for 3 h by adding 1.0 m*M* IPTG in the absence of hemin. The cells were then lysed by sonication and centrifuged. The supernatant was recovered and purified by immobilized metal-affinity chromatography (IMAC). The His-tag used to facilitate the purification by IMAC was cleaved using HRV3C protease and proteins were recovered in 50 m*M* Tris buffer pH 8 supplemented with 150 m*M* NaCl. Yields were usually good, with 40–50 mg protein being produced per litre of bacterial culture.

### 2.2. Heme incorporation

*Bm*Bfr was produced as an apoprotein. To incorporate its heme cofactor, the following method was used (inspired by Wang *et al.*, 2015 ▸): 2.5 ml *Bm*Bfr concentrated to 0.8 mg ml^−1^ was heated for 15 min at 60°C in the presence of hemin chloride. If needed, excess hemin was then removed by decreasing the pH to 3.5 and filtration at 0.2 µm. Heme incorporation was checked by UV–visible spectrometry.

### 2.3. Crystallization, data collection and processing

Batches of *Bm*Bfr produced in the absence of hemin (apo *Bm*Bfr) yielded useful crystals at pH 7.5 (0.1 *M* HEPES buffer) using 1.5 *M* lithium sulfate as a precipitating agent. For crystallization, the protein was concentrated to 10 mg ml^−1^ and the hanging-drop method was used with 0.5 µl enzyme solution mixed with 0.5 µl well solution (SG2 crystallization kit from Molecular Dimensions; Abrahams & Newman, 2021 ▸; Fazio *et al.*, 2014 ▸). Crystals took three days to grow in octahedral forms measuring about 100 µm.

Apo *Bm*Bfr crystals were cryoprotected with 20% glycerol and diffraction data were collected to a resolution of 1.97 Å on beamline PROXIMA-2A (100 K, wavelength of 0.97857 Å and EIGER X 9M detector) at Synchrotron SOLEIL, Gif-sur-Yvette, Paris (Duran *et al.*, 2013) ▸, using *MxCuBE* (Oscarsson *et al.*, 2019 ▸) for data collection and *XDS*/*XSCALE* (Kabsch, 2010 ▸) for processing. Molecular replacement was performed by *Phaser* (McCoy *et al.*, 2007 ▸) in *Phenix* (Liebschner *et al.*, 2019 ▸) using PDB entry 3fvb. *Phenix*, *Coot* (Emsley *et al.*, 2010 ▸) and *eLBOW* (Moriarty *et al.*, 2009 ▸) were used to build the model and refine the structure. The final coordinates were deposited as PDB entry 9gzu. Figures were generated using *PyMOL* (DeLano, 2002 ▸).

Data-collection and refinement statistics are given in Table 1 ▸ for the apo *Bm*Bfr structure.

### 2.4. *In silico* study of oligomeric state *viaPISA*

Study of the protein interfaces, surfaces and stability of the assemblies was performed using the *PDBePISA* web interface (Krissinel & Henrick, 2007 ▸). Atomic coordinates of both the holo and apo *Bm*Bfr crystal structures were analyzed.

### 2.5. Experimental study of the oligomerization state by size-exclusion chromatography (SEC)

Size-exclusion chromatography measurements were performed on an NGC Bio-Rad FPLC using a Cytiva Superdex 200 Increase 10/300 GL column. After column equilibration with an appropriate buffer, a 300 µl sample of protein at 6 mg ml^−1^ was loaded and eluted at 0.5 ml min^−1^. A calibration curve was performed in working buffer with Gel Filtration Markers Kit for Protein Molecular Weights 29 000–700 000 Da from Sigma–Aldrich, showing a good correlation between molecular weight (*M*_w_) and the ratio between elution volume and dead volume (*V*_e_/*V*_o_),
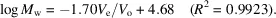


Good correlation was also obtained with the hydrodynamic radius (*R*_h_) determined by the *Hullrad* program (Fleming & Fleming, 2018 ▸),
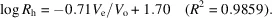


Stock solutions of *Bm*Bfr in the presence or absence of hemin at different pH values (50 m*M* sodium citrate or Tris, 0.150 *M* NaCl, pH 3.5–7.5) were analyzed to deduce their oligomeric states in solution. To determine the oligomeric states, the hydrodynamic radii were calculated using the calibration equation and compared with those predicted by the *Hullrad* program (Supplementary Table S1).

### 2.6. Protein stability studied by thermal shift assay

Thermal shift assays (TSAs) were performed using the StepOnePlus Real-Time PCR system from ThermoFisher Scientific. The wells of the PCR plate were filled with 20 µl of a solution containing 12 µg protein and the desired amount of the tested molecule, in the presence of SYPRO Orange from ThermoFisher Scientific (1× concentrated). Measurements of fluorescence at 623 nm (excitation at 570 nm) were performed every degree between 25 and 98°C. TSA curves were analyzed using *GraphPad Prism* 5.0 (GraphPad Software, La Jolla, California, USA) by fitting a Boltzmann sigmoid to the fluorescence transition.

## 3. Results

### 3.1. Crystal structure of *Bm*Bfr in the presence and absence of heme cofactor

**Figure 2:**
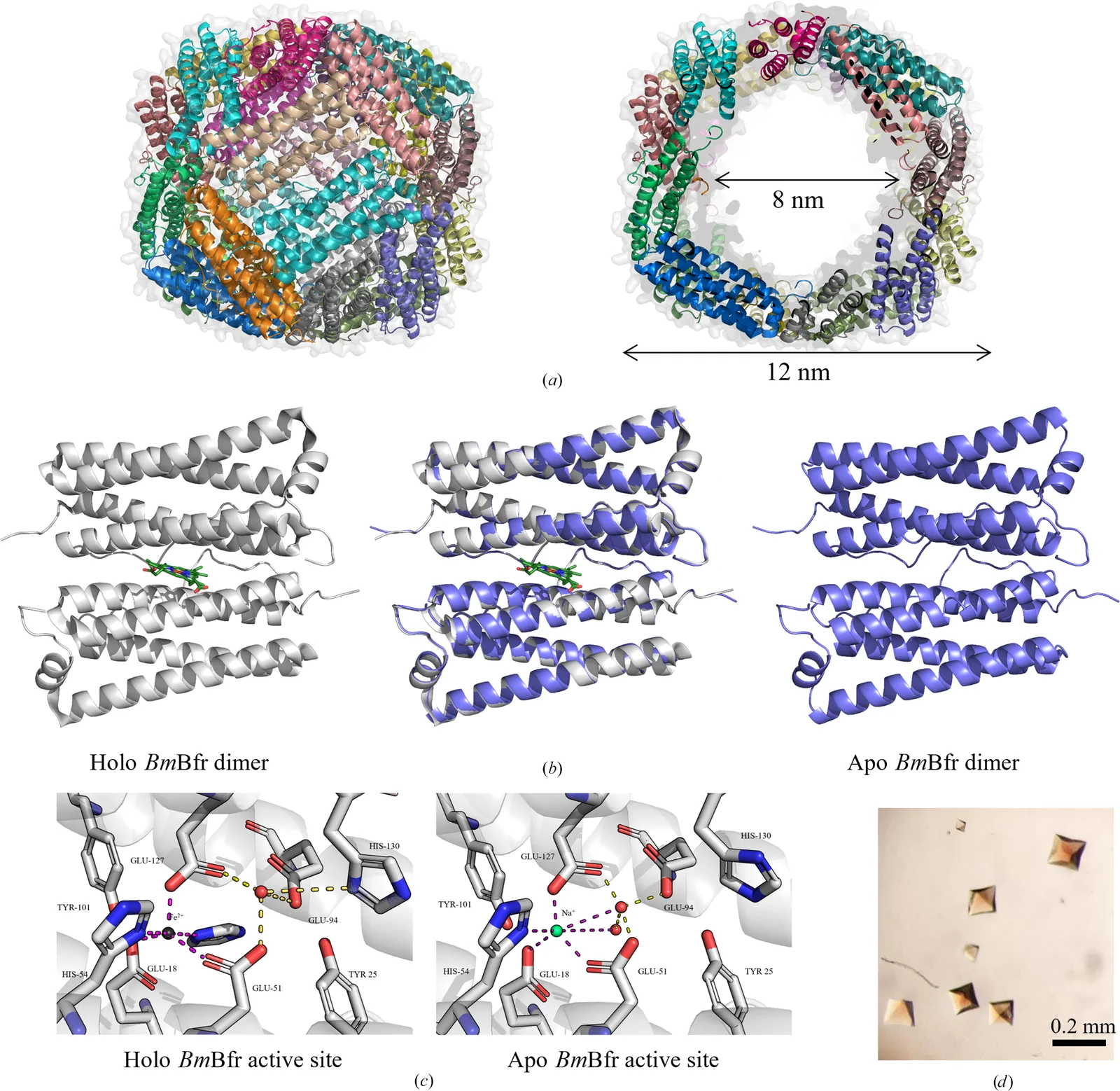
Crystal structures of *B. melitensis* bacterioferritin. (*a*) 24-subunit assembly creating a 12 nm outer diameter shell with an 8 nm interior cavity. (*b*) Comparison of the dimer accommodating the heme cofactor (green) in the holo *Bm*Bfr structure (left, white) or without cofactor (apo *Bm*Bfr, blue, right). An alignment of this interface in both structures is presented (middle). (*c*) Comparison of the ferroxidase centers in the holo *Bm*Bfr (left) or apo *Bm*Bfr structures (right). (*d*) Picture of apo *Bm*Bfr crystals.

The crystal structure of apo *Bm*Bfr determined in this work was compared with that of holo *Bm*Bfr (PDB entry 3fvb; Seattle Structural Genomics Center for Infectious Disease. unpublished work). *B. melitensis* bacterioferritin crystallizes, both in the apo and holo forms, as a 24-subunit protein that self-assembles into a spherical nanocage, adopting the classical ferritin fold observed in similar proteins (Arosio *et al.*, 2009 ▸; Dautant *et al.*, 1998 ▸; Frolow *et al.*, 1994 ▸; Granier *et al.*, 2001 ▸; Jobichen *et al.*, 2023 ▸; Lawson *et al.*, 1991 ▸; Précigoux *et al.*, 1994 ▸; Yao *et al.*, 2012 ▸). Each subunit has a four-helix bundle fold, and the 24 subunits assemble to create a 12 nm outer diameter shell with an 8 nm interior cavity (Fig. 2 ▸*a*).

In the presence of added hemin, holo *Bm*Bfr contains 12 heme cofactors bound at the interfaces between subunits related by twofold symmetry. In the absence of hemin, apo *Bm*Bfr adopts the same 24-mer oligomeric state but the heme cofactor is absent at the dimeric interfaces, as confirmed by careful analysis of electron density at this interface (polder maps; Supplementary Fig. S1). These differences do not induce major conformational changes between the structures, as underlined by an overall r.m.s.d. measured on all C^α^ atoms of 0.24 Å (Fig. 2 ▸*b*).

Another major difference is the presence of iron in the holo *Bm*Bfr crystal structure that is not present in the apo *Bm*Bfr structure (Fig. 2 ▸*c*).

Crystals of apo *Bm*Bfr analyzed by XRD showed a slight brown-red coloration (Fig. 2 ▸*d*) that could indicate the presence of heme in the protein structure (Eaton & Hochstrasser, 1968 ▸; Pfanzagl *et al.*, 2020 ▸; Rodrigues *et al.*, 2006 ▸). Nucleation points may have been formed through the crystallization of the holo form. The presence of holo *Bm*Bfr in apo solution observed by SEC strengthens this idea.

### 3.2. *In silico* analysis of the oligomeric states of *Bm*Bfr

*PISA* (*Protein Interfaces, Surfaces, and Assemblies*; Krissinel & Henrick, 2007 ▸) is a powerful and widely used tool for predicting protein oligomeric states from crystal structures. The program analyzes protein crystal structures to identify the most probable oligomeric state by assessing macromolecular interactions. It does this by calculating the Gibbs free energy of dissociation (Δ*G*°) for various potential assemblies and using this energy to predict which assemblies are stable in solution.

**Figure 3:**
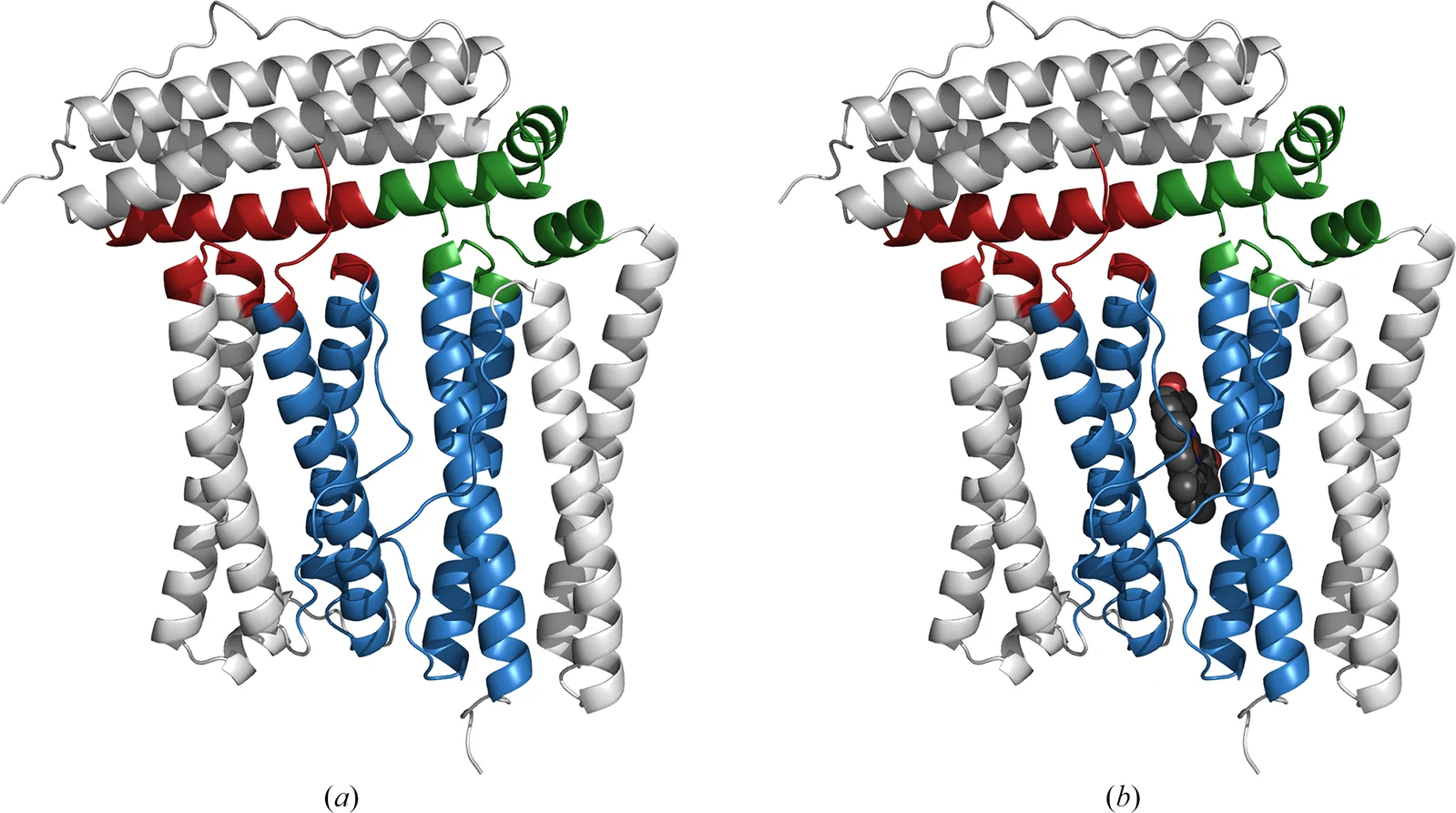
Primary interfaces of (*a*) apo and (*b*) holo *Bm*Bfr selected with *PISA*: interface 1 (dimeric interface) is in blue, interface 2 is in red and interface 3 is in green.

Three primary interfaces were found with *PISA*, as shown in Fig. 3 ▸. Table 2 ▸ provides characteristics of selected interfaces within the 24-mer oligomeric structures of apo and holo *Bm*Bfr.

**Figure 4:**
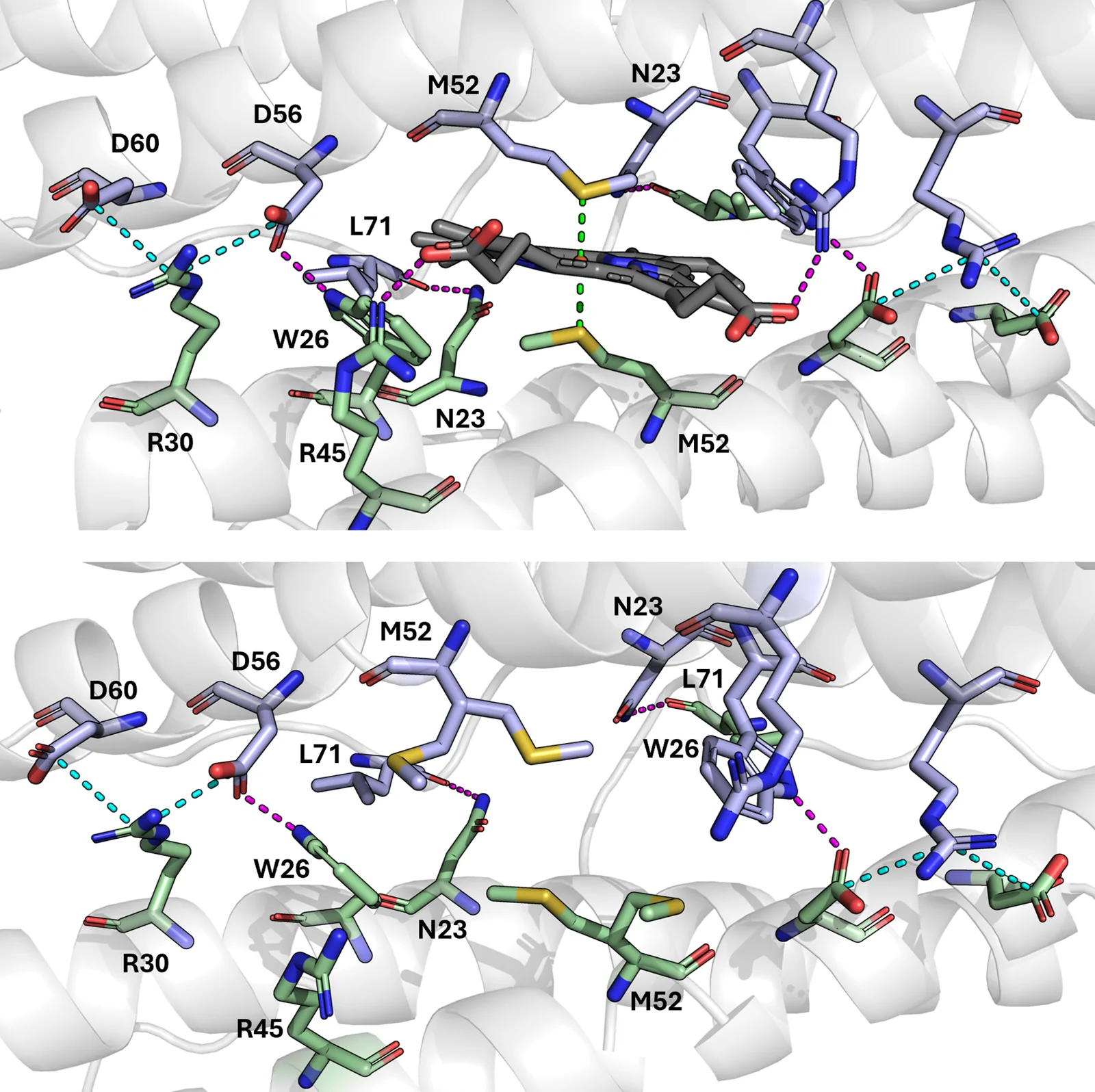
Selected interactions at the dimeric interface of holo (top) and apo (bottom) *Bm*Bfr.

In both structures, the dimeric interface (interface 1) is the most stable interface identified by *PISA*. The stability of this interface is associated with a series of interactions, including salt bridges between Arg30 and Asp60 and between Arg30 and Asp56. When occupied by the heme cofactor in holo *Bm*Bfr, additional interactions appear at the dimeric interface, including the coordination of the heme iron cation by Met52 and an additional salt bridge involving the carboxylate of the heme and Arg45 (Fig. 4 ▸). The stability of this interface is also associated with a negative solvation Δ*G*° (Table 2 ▸), which is more favorable in the presence of the heme cofactor.

Interfaces 2 and 3 display the same interactions and a similar solvation Δ*G*° for both apo and holo *Bm*Bfr, suggesting that the effect of heme is negligible for these interfaces. This observation is consistent with expectation, as the heme is distant from these interfaces (more than 16 Å).

### 3.3. Determination of oligomeric states in solution by size-exclusion chromatography

The oligomeric state of the protein can be affected by its environment. Crystallization occurs at a high protein concentration and with crystallization agents, leading to different oligomeric behavior to that in solution. Oligomeric interfaces predicted by *PISA* are then biased by the intrinsic crystal condition on which the analysis is based. Therefore, oligomeric states of *Bm*Bfr in solution were analyzed by size-exclusion chromatography to elucidate the effect of the aforementioned bias.

**Figure 5:**
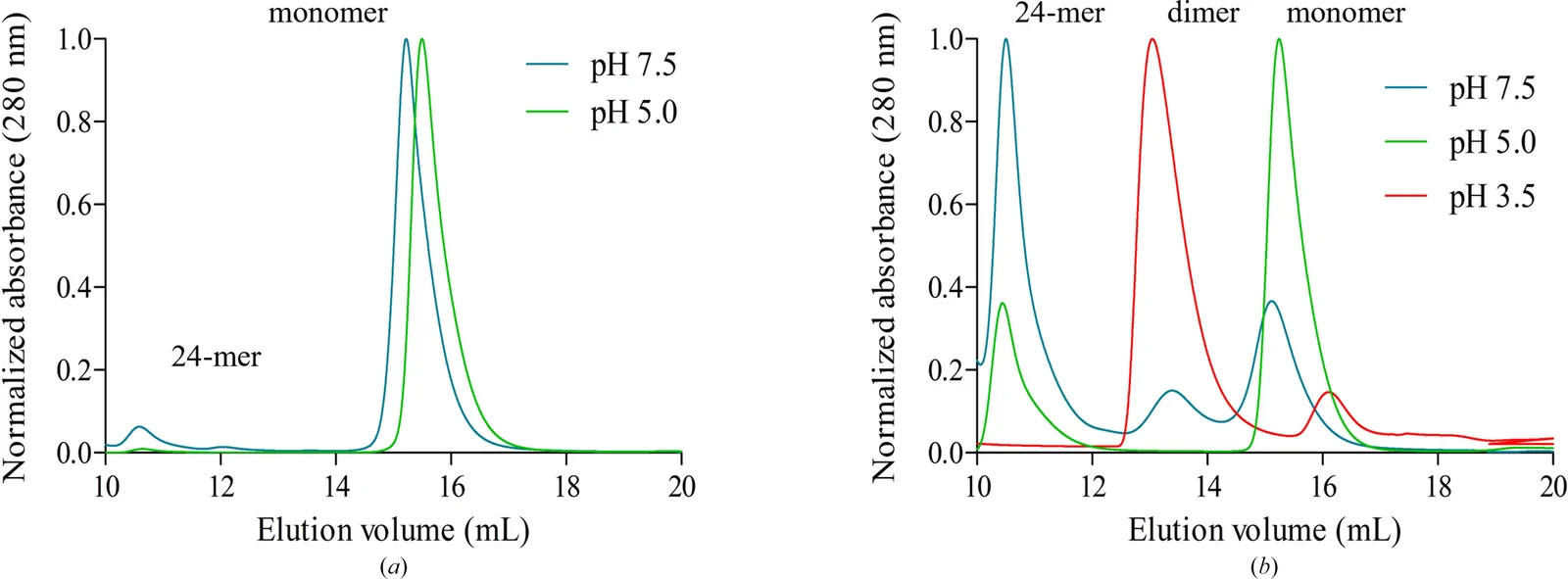
SEC analysis of apo *Bm*Bfr (*a*) and holo *Bm*Bfr (*b*) at different pH values. pH values of 3.5 and 5.0 were obtained with 50 m*M* citrate buffer and pH 7.5 with 50 m*M* Tris buffer. All buffers contain 250 m*M* NaCl.

In the absence of added heme in the solution, apo *Bm*Bfr eluted as a major peak corresponding to a monomer (Fig. 5 ▸*a*). The *R*_h_ measured by SEC (2.6 nm at pH 5 and 2.7 nm at pH 7.5) is indeed close to that predicted with *Hullrad* (2.4 nm; Supplementary Table S1). A neglectable portion of apo *Bm*Bfr eluted as a 24-mer. In the presence of added heme, the oligomeric state of holo *Bm*Bfr depended on the pH of the solution (Fig. 5 ▸*b*). Indeed, holo *Bm*Bfr eluted as a mixture of monomer (26%), dimer (7%) and 24-mer (67%) at pH 7.5. At pH 5.0, holo *Bm*Bfr exists as a mixture of monomer (74%) and 24-mer (26%). At lower pH (3.5) no traces of the 24-mer are observed, with holo *Bm*Bfr being essentially in the form of a dimer (92%) and traces of monomer (8%). The acidity of the solution thus has an important effect on the oligomeric state of the protein (Table 3 ▸).

Heme incorporation of the samples was checked by UV–visible absorbance to evaluate the Soret peak (Supplementary Fig. S3), confirming that the monomer is indeed heme-free while the dimer and 24-mer incorporate the cofactor.

### 3.4. Stability analysis of *Bm*Bfr by thermal shift analysis (TSA)

**Figure 6:**
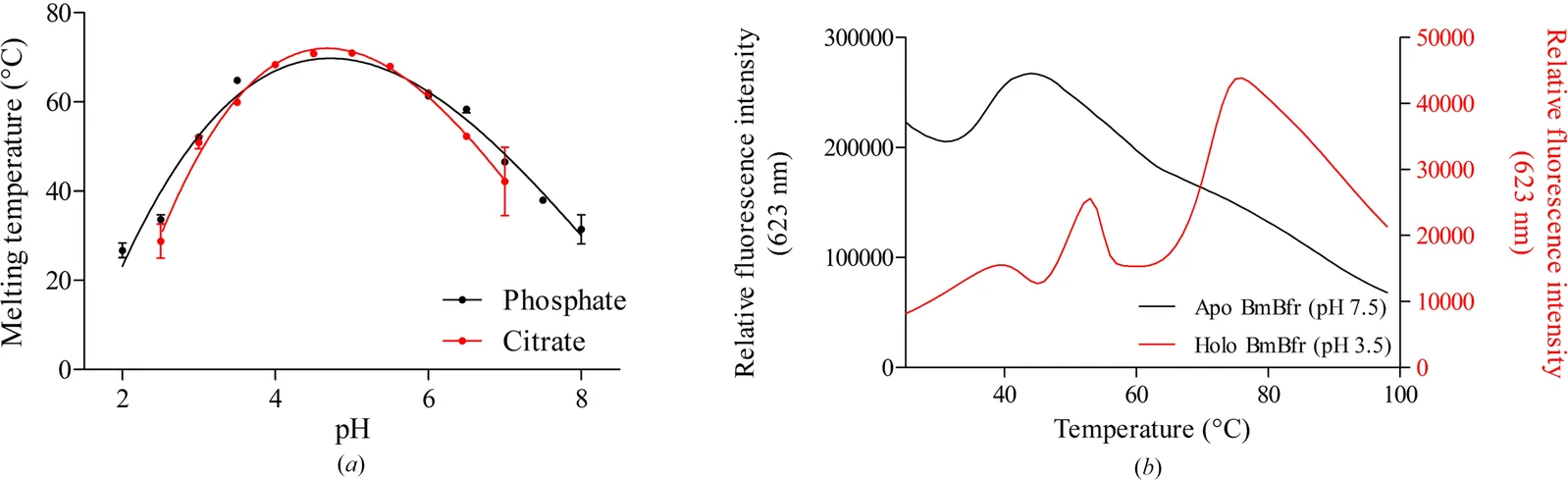
(*a*) Melting temperature of apo *Bm*Bfr at different pH values. Two buffers were used to fix the pH: phosphate (black) and citrate (red). (*b*) Thermal shift assay measurements for holo *Bm*Bfr in 50 m*M* citrate pH 3.5 [red, *T*_m_(1) = 49.6 ± 0.1°C, *T*_m_(2) = 70.5 ± 0.1°C] and apo *Bm*Bfr in 50 m*M* phosphate pH 7.5 (black, *T*_m_ = 38.0 ± 0.3°C).

*Bm*Bfr monomer stability was assessed over a wide range of pH values using the apoprotein (Fig. 6 ▸*a*). Two buffers were used to fix the pH (citrate and phosphate), showing a small effect of the buffer nature on protein stability. In both cases, maximum stability is achieved around pH 4.7. It corresponds to the protein isoelectric point, which was calculated to be 4.7 using *ProtParam* (Gasteiger *et al.*, 2005 ▸).

Thermal shift analysis was also performed to determine the stability of the 24-mer, but its exact melting temperature could not be assessed because of its great stability (*T*_m_ > 90°C at pH values between 4.0 and 8.0). This result is consistent with available information in the literature (Qu *et al.*, 2021 ▸). It was however possible to compare the dimer stability with that of the monomer at pH 3.5, where they are the only two states present in solution (Fig. 6 ▸*b*). The TSA curve of the dimer–monomer mixture at pH 3.5 (red) is compared with that for the monomer of the apoprotein at pH 7.5. Only one transition is observed in the monomer curve, while two are visible in the monomer–dimer curve, confirming the presence of two oligomeric states in solution. Based on the monomer curve and Fig. 6 ▸(*a*), the first transition can be ascribed to the monomer, showing stabilization of the dimer in solution (around 21°C more stable than the monomer).

## 4. Discussion

In this work, two crystal structures of *Bm*Bfr were studied, one obtained using the holo form (heme-containing form) and the other obtained using the apo form. The two structures are almost identical apart from the presence of heme at the dimeric interface in holo *Bm*Bfr and the composition of the ferroxidase center (Figs. 2 ▸*b* and 2 ▸*c*). Both forms crystallize in a face-centered cubic lattice, with similar unit-cell parameters but with different symmetry elements: holo *Bm*Bfr (PDB entry 3fvb) was crystallized in a less symmetric space group than apo *Bm*Bfr (PDB entry 9gzu).

To develop a deeper understanding of the distinctions between the structures, interfaces were studied with the *PISA* tool, highlighting three main interfaces in the 24-meric assemblies (Fig. 3 ▸). The first interface is the dimeric interface formed between two subunits related by twofold symmetry. This interface creates the heme-bonding pocket where the cofactor is present for the holo form only (Fig. 4 ▸). It is the most stable interface, and is even more stable in the presence of heme (Table 2 ▸), as previously observed for bacterioferritins from *E. coli* and *M. tuberculosis* (Le Vay *et al.*, 2020 ▸; Mohanty *et al.*, 2021 ▸).

This interface is the source of the main difference between apo and holo *Bm*Bfr. Specifically, in the absence of heme (apo *Bm*Bfr) this interface is not strong enough to allow dimerization in solution, as suggested by the SEC results, where only the monomer is observed at different pH values (Fig. 5 ▸*a*). However, after incubation with heme cofactor, the protein (holo *Bm*Bfr) oligomerizes to form a dimer, which was shown to be more stable than the monomer by TSA analysis at pH 3.5 (Fig. 6 ▸*b*). Depending on the pH conditions, dimers can oligomerize as a 24-mer, as revealed by the SEC results (Fig. 5 ▸*b*). These results suggest that dimer formation is essential for further oligomerization into 24-mers and that heme incorporation is essential for dimerization. This behavior is unexpected and contrasts with that of bacterioferritins from *E. coli*, *M.  tuberculosis* and *P. aeruginosa*, which show stable dimeric or 24-meric species even in the absence of heme cofactor (Andrews *et al.*, 1995 ▸; Mohanty *et al.*, 2021 ▸; Stein *et al.*, 2026 ▸). The requirement for heme in dimer assembly may constitute the evolutionary divergence between classical bacterioferritins and heme-deficient ferritin-like proteins. The heme group in *Bm*Bfr thus has three functions: facilitating electron transfer and iron release from the core, increasing structural stability and enabling oligomerization.

To further oligomerize into a 24-mer, two complementary interfaces are created between two different dimers (interfaces 2 and 3 in Fig. 3 ▸). These interfaces are completely conserved in the apo and holo *Bm*Bfr crystal structures. The formation of the 24-mer holo *Bm*Bfr leads to a very stable oligomeric state, with a transition above 90°C as observed by TSA. This high thermal stability of ferritins has previously been documented in the literature (Qu *et al.*, 2021 ▸).

The interfaces obtained for apo *Bm*Bfr are probably biased by the intrinsic crystallization conditions, as they are only observed in the solid crystalline state. Indeed, no oligomeric states higher than a monomer were observed in solution using SEC (Fig. 5 ▸*a*). The apo *Bm*Bfr crystal could then be obtained because of the high protein concentration and/or after the nucleation of holo *Bm*Bfr traces in the sample initiating crystallization, as suggested by the slightly brown-red crystal coloration (Fig. 2 ▸*d*).

Analyses were performed using SEC to further characterize holo *Bm*Bfr oligomerization in solution (Fig. 5 ▸*b*). Previous work in the literature for bacterioferritins from other species has shown variation in oligomeric states when going from highly acidic to neutral pH (Bradley *et al.*, 2022 ▸; Mohanty *et al.*, 2021 ▸). Considering this information, SEC measurements were performed at three different pH values (3.5, 5.0 and 7.5), showing three different oligomeric forms: monomer, dimer and 24-mer. Only the dimer and monomer are observed at pH 3.5, highlighting that this condition prevents 24-mer formation, probably by the destabilization of interfaces 2 and 3 at low pH. This destabilization is linked to the protonation of acidic residues involved in saline bridges and hydrogen bonding such as Asp34, Glu106, Glu128 and Glu174. At pH 5.0 only the 24-mer and monomer are observed, suggesting that the dimer–24-mer equilibrium is probably shifted to the 24-mer state. This is not surprising since the pH is close to the isoelectric point, promoting protein–protein interactions (Zhang *et al.*, 2011 ▸). Finaly, all states are visible at pH 7.5, at which the 24-mer can be formed but with weaker protein–protein interactions (at interfaces 2 and 3), thus coexisting with the dimer. The presence of the monomer at pH 5.0 and 7.5 is probably due to incomplete heme incorporation of the sample.

**Figure 7:**
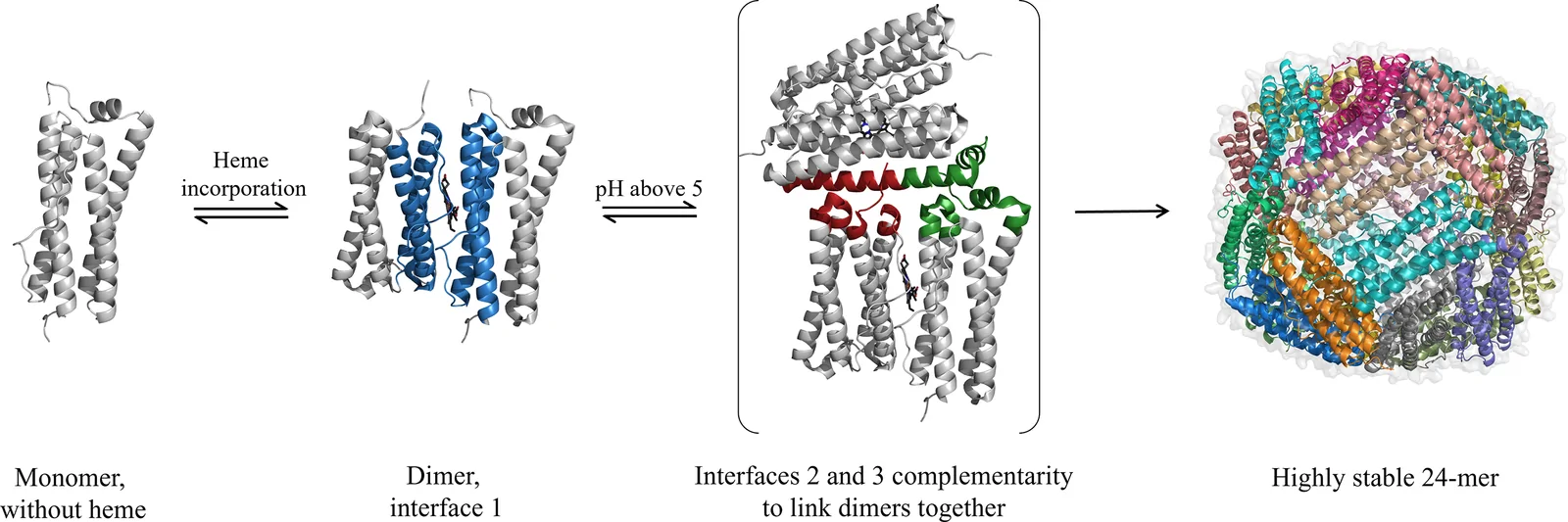
Oligomerization mechanism of *Bm*Bfr. The first step is ruled by heme incorporation to form the dimer, stabilized by highly favorable interactions in interface 1 (blue). Dimers then assemble into a higher oligomeric state using complementary interactions in interfaces 2 and 3 (red and green), above pH 5, to form the 24-mer.

Altogether, the different results obtained by crystallo­graphy, interface analysis, SEC and TSA lead to the suggestion of a complex oligomerization mechanism ruled by heme incorporation and pH regulation (Fig. 7 ▸).

Additional computational analyses could validate the actual mechanism of oligomeric disruption with greater confidence. They could include energy analysis to analyze hydrogen bonds (short range/long range/backbone/side chain), van der Waals interactions and electrostatic interactions to obtain clarity on the mechanism of stabilization. Constant pH replica exchange molecular dynamics (CpHMD; Mongan *et al.*, 2004 ▸; Swails *et al.*, 2014 ▸) could also be carried out for both apo and holo forms of *Bm*Bfr. Unlike standard MD, which uses fixed protonation states, CpHMD allows titratable residues (*e.g.* His, Asp snd Glu) to change protonation states during the simulation based on the surrounding environment and the target pH, enabling the study of pH-dependent structural changes and binding. This would further help to understand the transition in hydrogen-bond and other chemical interactions during oligomer disruption and complement the experimental evidence provided in the present work.

## Supplementary Material

PDB reference: apo bacterioferritin from *Brucella melitentsis*, 9gzu

Supplementary Figures and Table. DOI: 10.1107/S2059798326006625/jb5073sup1.pdf

## Figures and Tables

**Table 1:** Data-collection and refinement statistics for apo *Bm*Bfr Values in parentheses are for the highest resolution shell.

PDB code	9gzu
Wavelength (Å)	0.97857
Resolution range (Å)	43.44–1.97 (2.04–1.97)
Space group	*F*432
*a,b*, *c* (Å)	173.78, 173.78, 173.78
α, β, γ (°)	90, 90, 90
Total reflections	1263320 (125430)
Unique reflections	16468 (1585)
Multiplicity	76.7 (79.1)
Completeness (%)	99.95 (99.94)
Mean *I*/σ(*I*)	36.62 (4.67)
Wilson *B* factor (Å^2^)	27.47
*R*_merge_	0.1487 (1.363)
*R*_meas_	0.1497 (1.372)
*R*_p.i.m._	0.01709 (0.1531)
CC_1/2_	1 (0.946)
CC*	1 (0.986)
Reflections used in refinement	16465 (1585)
Reflections used for *R*_free_	823 (78)
*R*_work_	0.1977 (0.2092)
*R*_free_	0.2353 (0.2752)
CC_work_	0.936 (0.928)
CC_free_	0.908 (0.894)
No. of non-H atoms	
Total	1515
Macromolecules	1326
Ligands	19
Solvent	170
Protein residues	162
R.m.s.d., bond lengths (Å)	0.008
R.m.s.d., angles (°)	0.83
Ramachandran plot	
Favored (%)	98.75
Allowed (%)	1.25
Outliers (%)	0.00
Rotamer outliers (%)	0.72
Clashscore	4.91
Average *B* factor (Å^2^)	
Overall	27.82
Macromolecules	26.45
Ligands	38.32
Solvent	37.30

**Table 2:** Primary interfaces characterization of apo and holo *Bm*Bfr: interface area, solvation Δ*G* and number of hydrogen bonds, salt bridges and coordination bonds

	Structure (PDB code)	Interface area (Å^2^)	Δ*G*^solvation^ (kcal mol^−1^)	Hydrogen bonds	Salt bridges	Coordination bonds
Interface 1	Holo *Bm*Bfr (3fvb)	959.3	−10.8 and −8.7†	6	4	2
	Apo *Bm*Bfr (9gzu)	986.7	−12.9	4	4	0
Interface 2	Holo *Bm*Bfr (3fvb)	467.2	−2.8	4	1	0
	Apo *Bm*Bfr (9gzu)	487.7	−3.9	4	1	0
Interface 3	Holo *Bm*Bfr (3fvb)	626.1	−5.2	4	1	0
	Apo *Bm*Bfr (9gzu)	659.4	−5.0	4	1	0

† Δ*G*^solvation^ for the monomer–monomer interface and mean Δ*G*^solvation^ for monomer–heme interfaces, respectively.

**Table 3:** Percentage of the holo *Bm*Bfr oligomeric states observed at different pH values

pH	24-mer (%)	Dimer (%)	Monomer (%)
7.5	67	7	26
5.0	26	0	74
3.5	0	92	8
