# Supplementary material for: Influence of heme and pH on the oligomeric states of Brucella melitensis bacterioferritin
Source: Acta Crystallogr D Struct Biol. 2026 Aug 10;82(Pt 9):1062–71. doi: 10.1107/S2059798326006625 (PMC13532695; doi:10.1107/S2059798326006625)
Supplement: Supplementary file 1 [file d-82-01062-sup1.pdf]

STRUCTURAL BIOLOGY

Volume 82 (2026)

Supporting information for article:

Influence of heme and pH on the oligomeric states of *Brucella melitensis* bacterioferritin

Tanguy Scaillet, Clément Champagne and Johan Wouters

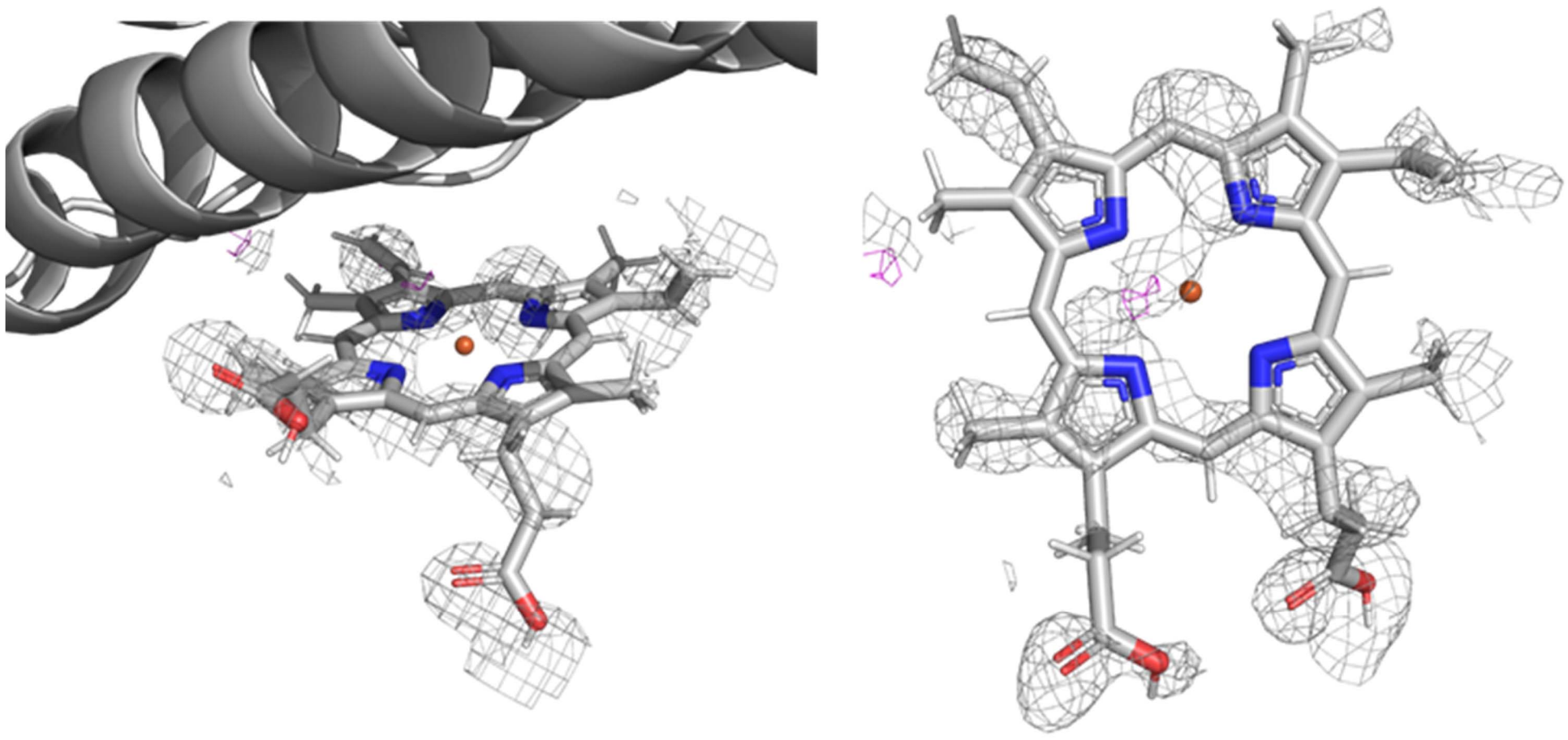

Figure S1 Electron density – $2F_o$-F_c map in gray (σ=1.0, carve=1.6) and polder density map in magenta (σ=3.0, carve=2.3) – showing that heme is not present in the structure.

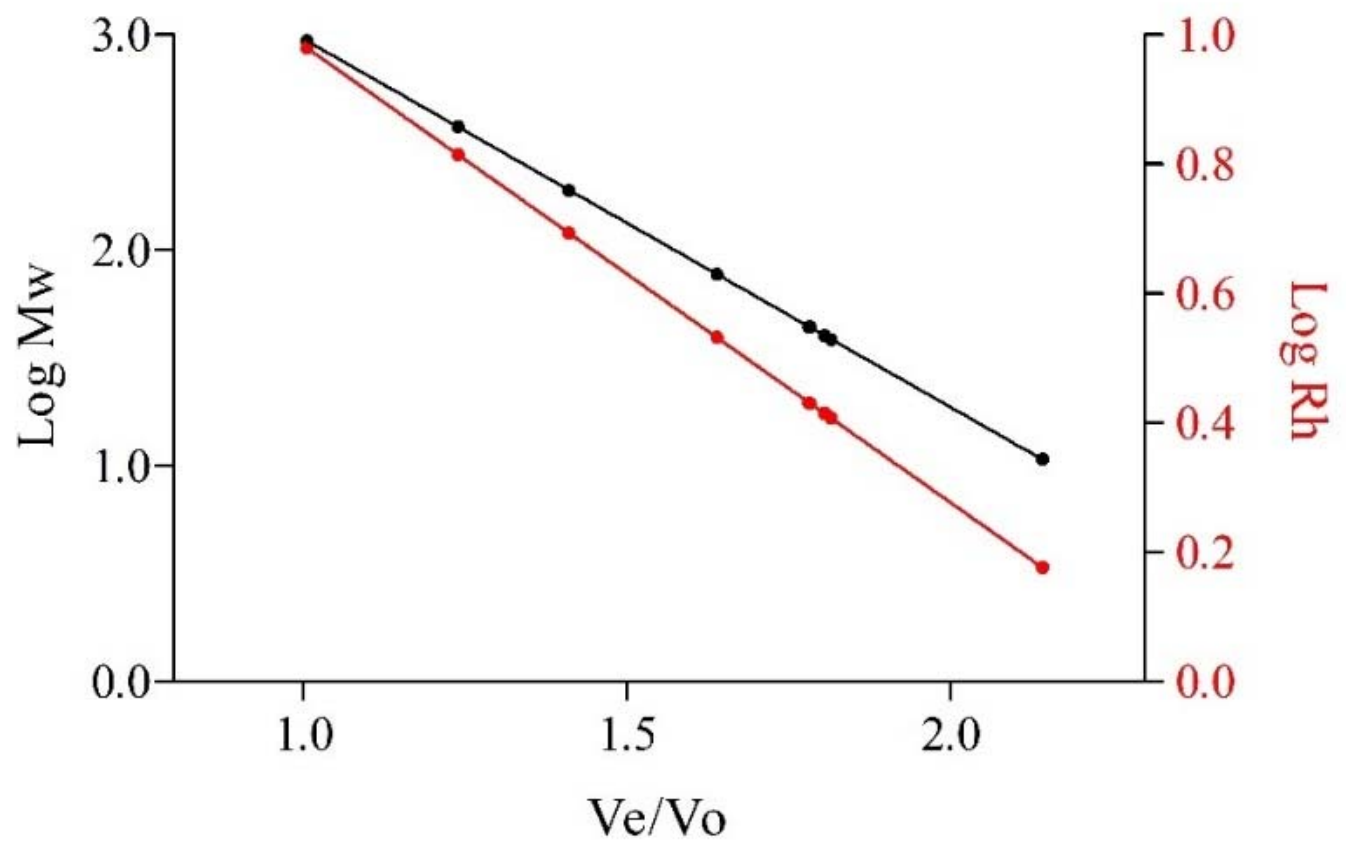

Figure S2 Linear regression for column calibration. Black: log Mw = -1.70 Ve/Vo + 4.68 (R^2 = 0.9923). Red: log Rh = -0.71 Ve/Vo + 1.70 (R^2 = 0.9859).

Table S1 Molecular weight, hydrodynamic radii calculated by HullRad, and predicted elution volume based on Rh for different oligomeric states.

	Molecular weight (kDa)	Hydrodynamic radius (nm)	Predicted elution volume (mL)
Monomer	18.9	2.4	15.8
Dimer	37.7	3.0	14.7
12-mer	226.4	5.5	11.5
24-mer	452.8	6.4	10.7

A

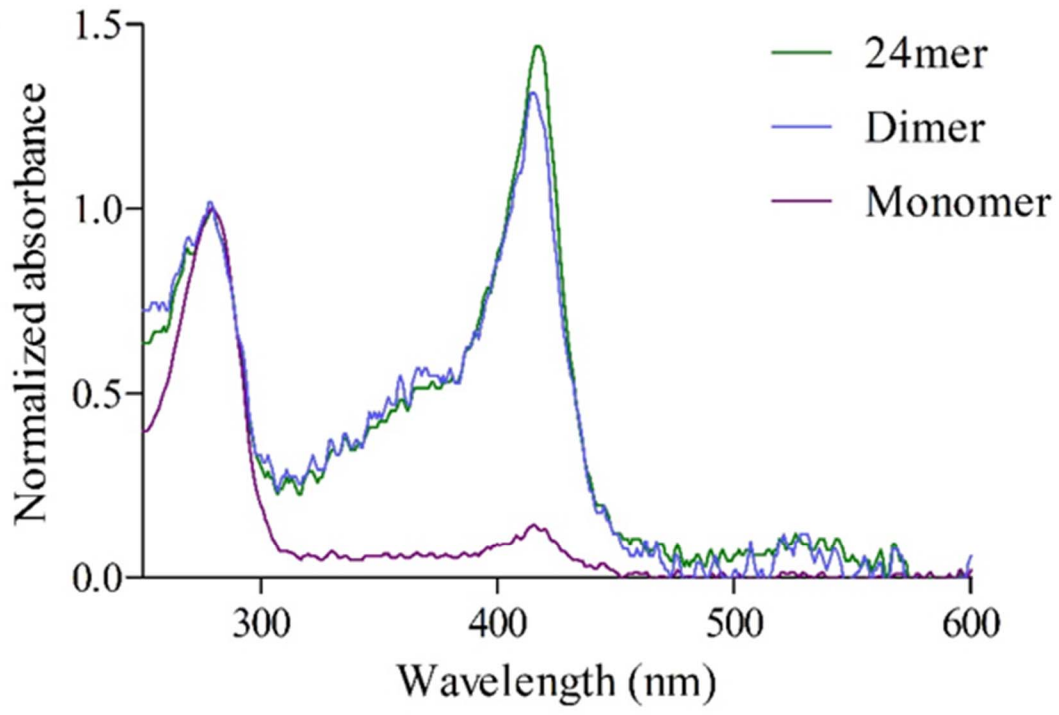

B

	$\frac{A_{heme}(417\ nm)}{A_{protein}(280\ nm)}$
24mer	1.44
Dimer	1.31
Monomer	0.14

Figure S3 A) Normalized absorbance spectra of sample before heme incorporation (purple, before SEC in figure 5A) and after heme incorporation (green and blue, fraction respectively collected for the 24mer and dimer after SEC in figure 5B). B) Ratios of heme group absorbance to protein absorbance.
